# Biopolymeric Scaffolds with Melatonin for Tissue Engineering—A Review

**DOI:** 10.3390/ijms26062520

**Published:** 2025-03-11

**Authors:** Beata Kaczmarek-Szczepańska, Sylwia Grabska-Zielińska

**Affiliations:** 1 Laboratory for Functional Polymeric Materials, Faculty of Chemistry, Nicolaus Copernicus University in Torun, Gagarin 7, 87-100 Toruń, Poland; 2 Faculty of Chemical Technology and Engineering, Bydgoszcz University of Science and Technology, Seminaryjna 3, 85-326 Bydgoszcz, Poland; sylwia.grabska-zielinska@pbs.edu.pl

**Keywords:** melatonin, biopolymers, scaffolds, tissue engineering

## Abstract

Melatonin, a natural hormone with antioxidant, anti-inflammatory, and regenerative properties, has gained increasing attention in tissue engineering for its ability to enhance the therapeutic potential of biopolymeric scaffolds. These scaffolds, designed to mimic the extracellular matrix, provide structural support and a bioactive environment for tissue regeneration. By integrating melatonin, researchers aim to create multifunctional scaffolds that promote cell proliferation, modulate inflammatory responses, and improve wound healing outcomes. Challenges in utilizing melatonin include maintaining its stability under light, heat, and oxygen exposure, and optimizing its release profile for sustained therapeutic effects. Innovative fabrication methods, such as electrospinning, 3D printing, and lyophilization, have enabled precise control over scaffold architecture and melatonin delivery. These techniques ensure enhanced interactions with target tissues and tailored regeneration processes. Combining melatonin with growth factors, cytokines, and antimicrobial agents offers the potential for multifunctional applications, from chronic wound management to bone and nerve regeneration. Continued research in this field promises transformative solutions in regenerative medicine, expanding the clinical applicability of melatonin-enriched scaffolds. This review highlights the current progress, challenges, and opportunities associated with harnessing melatonin’s therapeutic potential within tissue engineering frameworks.

## 1. Introduction

Biopolymeric scaffolds are indispensable in tissue engineering and regenerative medicine, offering a structural framework that mimics the natural extracellular matrix (ECM) of tissues [1,2,3,4,5]. These scaffolds provide a three-dimensional microenvironment that promotes cell adhesion, proliferation, and differentiation—key processes in tissue regeneration [6]. By facilitating cellular attachment, migration, and nutrient exchange, scaffolds create a supportive niche for tissue repair and healing [7]. In regenerative medicine, the ultimate aim is to fabricate bioactive substitutes that can effectively replace damaged tissues or organs [5,7,8]. Biopolymeric scaffolds not only offer mechanical support but also serve as temporary matrices guiding cell-driven tissue formation, with the scaffold degrading over time as new tissue replaces it [4].

The structural and functional properties of biopolymeric scaffolds are central to their success. Highly porous structures with interconnected pores enable efficient vascularization and nutrient exchange, which are essential for cell viability and tissue integration [9]. Pore size and distribution influence the type of tissue that can regenerate; for instance, larger pores are ideal for bone tissue engineering due to the need for vascularization, whereas smaller pores suit soft tissue or skin regeneration [10,11,12]. Similarly, the mechanical properties must align with the specific tissue application: bone scaffolds require rigidity for load-bearing, while scaffolds for cartilage or skin regeneration demand flexibility and elasticity to accommodate movement.

Biopolymeric scaffolds can be further functionalized to enhance cellular interactions. Biochemical cues, such as integrin-binding motifs and cell adhesion molecules, can be incorporated to mimic the ECM and guide cell behavior [13,14,15,16]. Moreover, scaffolds can be bioengineered to deliver therapeutic agents like growth factors, cytokines, or hormones, including melatonin. Melatonin’s incorporation brings additional benefits, leveraging its antioxidant, anti-inflammatory, and wound-healing properties to stimulate cell differentiation and tissue formation [17,18].

Recent studies have explored the potential use of biopolymeric scaffolds containing melatonin in tissue engineering applications [19,20]. These three-dimensional structures, composed of various biopolymers such as collagen, chitosan, hyaluronic acid, and polycaprolactone, mimic the extracellular matrix and enable the controlled release of melatonin. Furthermore, melatonin-loaded scaffolds have demonstrated biocompatibility and have been shown to promote rapid tissue regeneration [18,19,21,22,23]. Given the promising applications of melatonin-loaded biopolymeric scaffolds in tissue engineering and wound-healing therapies, this review has been proposed. To date, no comprehensive literature review has been published on biopolymeric scaffolds incorporating melatonin for tissue engineering applications. Existing reviews primarily focus on specific aspects, such as 3D printing strategies for polymeric materials loaded with melatonin in bone tissue regeneration [21], nanocarriers for melatonin delivery [24,25], or melatonin/sericin patches for melanoma treatment [26]. Therefore, our review aims to bridge this gap by providing an overview of the preparation methods for melatonin-loaded scaffolds and their key properties. Additionally, it briefly discusses the challenges and future perspectives in this emerging field.

Biopolymeric scaffolds offer significant benefits in tissue engineering due to their natural biocompatibility and ability to support cell adhesion, proliferation, and tissue regeneration [1]. Their biodegradable nature reduces the need for surgical removal, and they can be engineered to deliver bioactive molecules, enhancing healing processes. Additionally, their properties can be tailored through modifications, making them adaptable for various biomedical applications [27].

However, they also present some challenges, such as mechanical weakness, which limits their use in load-bearing tissues. Rapid degradation can sometimes compromise their function before full tissue regeneration occurs, while batch-to-batch variability in natural polymers can affect reproducibility. Structural stability issues, such as swelling or shrinkage, may impact performance, and in some cases, certain biopolymers may trigger immune responses [2,28,29]. Despite these drawbacks, advancements in scaffold engineering, including composite materials and hybrid structures, continue to improve their effectiveness in regenerative medicine.

## 2. Biofunctionalization of Scaffolds for Tissue Engineering

A significant advancement in scaffold technology lies in the integration of drug delivery systems, enabling scaffolds to act as reservoirs for therapeutic agents with controlled release over time. Biopolymeric scaffolds have shown remarkable potential in this area, particularly when incorporating bioactive compounds that enhance antioxidant [30], anti-inflammatory [31], and regenerative properties [32,33], supporting tissue healing and repair processes [4].

Biofunctionalization can involve embedding growth factors that stimulate cell proliferation, vascularization, and tissue regeneration [33]. These compounds not only enhance cellular activity but also create a dynamic microenvironment that mimics natural tissue development processes [34,35,36]. Growth factors play a pivotal role in guiding cellular behavior, including differentiation, migration, and matrix synthesis, ensuring that the scaffold supports the natural healing cascade [37]. For instance, in bone tissue engineering, growth factors promote osteogenic differentiation, facilitating the formation of new bone tissue and the integration of the scaffold with surrounding native bone. This is particularly critical in cases of large bone defects, where natural repair processes may be insufficient [38,39]. Similarly, in vascularization, growth factors stimulate the formation of new blood vessels, ensuring the delivery of oxygen and nutrients to regenerating tissues, which is essential for sustaining cell viability and functionality within the scaffold [40,41].

The incorporation of growth factors into scaffolds can also be tailored for specific applications by controlling their release kinetics [42]. The sustained or sequential release of growth factors can mimic the temporal aspects of natural tissue healing, ensuring that the right signals are present at the right time to guide regeneration [43,44]. This controlled release is often achieved through advanced fabrication techniques, such as encapsulation within nanoparticles or embedding in hydrogel matrices, allowing the precise regulation of bioactive agent availability [45,46,47]. In addition to growth factors, biofunctionalization may involve the use of signaling molecules and cytokines to modulate immune responses, reducing inflammation while promoting tissue repair [48,49,50]. By integrating multiple bioactive agents, scaffolds can address the complex interplay of biological processes involved in tissue regeneration, further enhancing their effectiveness [51]. The versatility of biofunctionalization not only broadens the scope of scaffold applications but also underscores their potential to address challenges in regenerative medicine, from accelerating healing in chronic wounds to enabling functional recovery in complex tissue systems [52,53,54].

Incorporating antimicrobial agents into scaffolds, such as silver nanoparticles [55], chlorhexidine [56], zinc oxide [57], or tannic acid [58], further enhances their functionality by preventing infections, which can compromise the healing process. These additions are particularly valuable in wound healing and other applications where infection risks are high [59]. Similarly, anti-inflammatory agents can modulate the immune response, reducing inflammation and creating a favorable environment for tissue regeneration. These agents play a critical role in mitigating the chronic inflammatory response that can hinder healing, ensuring balanced and regulated immune activity at the site of injury. Incorporating such agents into scaffolds not only promotes faster recovery but also minimizes the tissue damage caused by prolonged inflammation. Examples of the anti-inflammatory agents used in scaffolds include curcumin [60], resveratrol [61] and dexamethasone [62].

Scaffolds may also be enhanced with antioxidants to protect regenerating tissues from oxidative stress, improving cell survival and function. Such functionalization not only accelerates the healing process but also ensures a more robust and sustainable regeneration outcome [6,52,63]. These integrated systems represent a significant step forward in scaffold technology, offering tailored solutions for diverse regenerative medicine applications [64]. The inclusion of melatonin within biopolymeric scaffolds not only supports regenerative processes but also contributes to creating a biologically active microenvironment [23]. By regulating oxidative stress and inflammatory responses, melatonin can optimize cellular activities such as proliferation and differentiation [65]. Studies have shown that scaffolds infused with melatonin improve the healing outcomes of critical-sized bone defects and support soft tissue regeneration [18]. These findings highlight melatonin’s dual role in scaffolds: as a therapeutic agent and as a mediator of cellular responses within the tissue engineering framework.

## 3. Melatonin in Tissue Engineering

Melatonin (Mel), a naturally occurring hormone, has gained attention for its regenerative properties when incorporated into biopolymeric scaffolds. It possesses antioxidant, anti-inflammatory, and pro-osteogenic (bone-forming) activities that make it highly beneficial for tissue engineering applications [66]. By incorporating melatonin into biopolymeric scaffolds, researchers can create materials that not only provide structural support for tissue regeneration but also actively promote healing at a molecular level. Melatonin can be embedded in the scaffold matrix and released in a controlled manner, ensuring that the therapeutic effects are sustained over time, which is crucial for long-term tissue repair processes [20].

Melatonin helps reduce oxidative stress in damaged tissues, protecting cells from free radicals and enhancing their survival and proliferation. This is particularly important in healing wounds or regenerating tissues in environments with high oxidative damage, such as after injury or surgery [17,67]. Melatonin also modulates the immune response by reducing pro-inflammatory cytokines and promoting a balanced inflammatory response, which is essential for preventing chronic inflammation and promoting tissue repair [68,69,70]. Melatonin has been shown to enhance osteoblast differentiation and mineralization, making it especially useful in bone tissue engineering. By promoting the formation of bone matrix, melatonin-enriched scaffolds can accelerate bone regeneration [71,72,73,74].

In skin regeneration, melatonin accelerates wound closure by promoting keratinocyte and fibroblast proliferation, enhancing collagen production, and modulating the inflammatory response [75,76]. This multifaceted activity of melatonin addresses several critical aspects of the wound healing process, making melatonin-enriched scaffolds particularly effective for treating complex conditions such as chronic wounds, diabetic ulcers, and burns.

Melatonin’s potent antioxidant properties help neutralize reactive oxygen species (ROS), which are commonly elevated in injured tissues, thereby reducing oxidative stress and preventing further damage to surrounding cells [67,77,78]. Additionally, melatonin regulates the expression of pro-inflammatory cytokines, such as tumor necrosis factor-alpha (TNF-α) and interleukin-6 (IL-6), while simultaneously promoting the release of anti-inflammatory mediators [79]. This dual modulation ensures a balanced immune response, preventing prolonged inflammation that can delay healing.

Another critical advantage of melatonin in skin regeneration is its ability to enhance angiogenesis—the formation of new blood vessels. By promoting the expression of vascular endothelial growth factor (VEGF) and other pro-angiogenic factors, melatonin supports the vascularization of regenerating tissues [80]. This improves oxygen and nutrient delivery to the wound site, which is essential for sustaining cellular activity and facilitating rapid tissue repair.

## 4. Preparation of Scaffolds Containing Melatonin 

The combination of polycaprolactone (PCL), polylactide (PLA), chitosan (CTS) or their blends with other polymers has been extensively studied in conjunction with melatonin. Various fabrication techniques have been employed and reported depending on the specific polymer system. For instance, the 3D multilayer molding method [81], solvent casting, the particulate leaching method with the entrapment of melatonin nanoparticles (MNP) [82], 3D printing [83,84], electrospinning [20,66,85,86], and lyophilization [22,23,87,88,89] have been used (Table 1).

Three-dimensional multilayer molding is a simple method in which polymeric components, as well as active substances, have to be dissolved in an appropriate solvent and injected into the jet sprayer. The solution is then sprayed onto the mold. Using this method, scaffolds of various shapes can be fabricated, as the polymer solution can be sprayed onto molds of different geometries. Quian et al. [81] utilized a tubular mold with microneedles to create scaffolds intended for sciatic nerve regeneration. The microneedles facilitated the development of a multiporous structure, enabling the exchange of proteins, water, and other nutrients. This feature was particularly advantageous when the scaffolds were applied as biomaterials for sciatic nerve repair in a rat model. Three-dimensional multilayer molding is a technique that allows excellent quality control, strong mechanical support, the alignment of gaps between nanofibers, and the even distribution of melatonin delivery [81].

Solvent casting and the particulate leaching method with the entrapment of melatonin nanoparticles (MNP) allowed the creation of interlinked porous scaffolds, where loaded melatonin was amorphized and remained chemically inert to the fabrication process [82].

Three-dimensional (3D) printing is an advanced additive manufacturing technology that has emerged as a cutting-edge approach to anatomical modeling, the creation of artificial tissues and organs, grafting, and tissue bridging. On the other hand, 3D printing technology is a precise, fast, and highly controllable fabrication method that aligns perfectly with the demands of personalized medicine [21,84]. Its versatility has led to widespread applications across various fields, including tissue engineering. In this context, 3D printing has emerged as one of the fastest-growing techniques for scaffold fabrication. This next-generation additive manufacturing approach is widely utilized for anatomical modeling, the production of artificial tissues and organs, as well as grafting and bridging damaged tissues, offering innovative solutions for regenerative medicine [21,84].

Electrospinning is a technique widely used for preparing biological scaffolds, but the selection of appropriate spinning materials remains a critical challenge. One solution to this issue involves using a single polymer (e.g., polycaprolactone or polylactide) blended with another, such as gelatin, to enhance the material properties [85,86]. Another approach is to prepare the scaffold via electrospinning (e.g., based polycaprolactone) and subsequently coat it with a hydrogel made from a different biopolymer, such as sodium alginate [20]. In both cases, the combination of two polymers improves the properties of the scaffold. Notably, melatonin can be incorporated into such scaffolds through the electrospinning mixture.

Lyophilization, or freeze-drying, is a very popular and simple method used to prepare porous scaffolds based on chitosan, collagen, alginate or other biopolymers enriched with bioactive substances for tissue engineering [22,23,85,87,88,89]. The freeze-drying process involves freezing a solution and subjecting it to vacuum drying, which removes water and solvents through sublimation. As water leaves the frozen polymer, interconnected pores are formed within the structure. This is a processing method that operates at low temperatures, enabling the incorporation of natural polymers and heat-sensitive biomolecules like melatonin. A limitation of freeze-drying is that the residual solvents used to dissolve the polymer or active substance may remain in the material after the process. Additionally, irregularities in porosity can also occur. The resulting scaffolds can achieve porosities above 90% and pore sizes ranging from 20 to 400 μm [90].

Combined preparation methods have also been used to obtain scaffolds enriched with melatonin. For example, melatonin-loaded gelatin sponges were obtained by foaming together with lyophilization [91]. Tween 80 has been used as a foaming agent. The components of sponges (gelatin and melatonin) have been mixed with a foaming agent and cross-linker, frozen, and lyophilized to obtain porous materials with an internal porous structure [91].

**Table 1:** Summary of melatonin-based biomaterials—their preparation methods, matrix and application.

Processing Method	Matrix	Application	References
3D multilayer molding method	Polycaprolactone	sciatic nerve regeneration	[81]
Solvent casting and particulate leaching method with MNP entrapment	Polycaprolactone	cartilage regeneration	[82]
3D printing	Magnesium/polycaprolactone	inhibition the development of osteosarcoma	[84]
	Polycaprolactone/β-TCP/ PLGA NPs	diabetic bone defect repairing	[83]
Electrospinning	Polycaprolactone/alginate	sciatic nerve regeneration	[92]
	Polycaprolactone/ sodium alginate	tendon regeneration	[20]
	Polycaprolactone/gelatin	nerve tissue engineering	[86]
	Gelatin/polylactide	vascularized bone regeneration	[85]
Lyophilization	Alginate/chitosan/β-TCP	tibia bone defect regeneration	[89]
	Alginate/chitosan/β-TCP	periodontal regeneration	[88]
	Chitosan/hydroxyapatite	bone formation and inhibiting osteoclast activity	[87]
	Chitosan/hydroxyapatite	osteosarcoma therapy	[93]
	Chitosan/ hydroxyapatite/PLGA NPs	bone formation and inhibiting osteoclast activity	[94]
	Chitosan/collagen	wound treatment	[22,23]
Combined method: foaming and lyophilization	Gelatine	skin tissue engineering	[91]

β-TCP—β-Tricalcium phosphate; NPs—nanoparticles; PLGA—poly(lactic-co-glycolic).

## 5. Properties of Scaffolds’ Containing Melatonin

**Figure 1:**
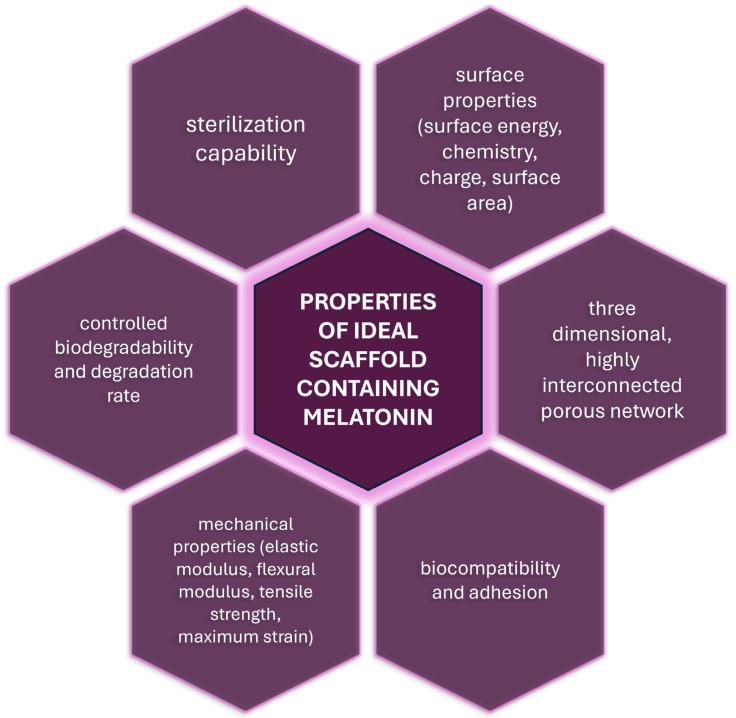
The properties of an ideal scaffold containing melatonin.

Polymeric scaffolds serve as carriers for cells and inductive factors, and play an active role in instructing cells and providing step-by-step guidance for tissue formation. To achieve this functionality, a deep understanding of the chemistry and physicochemical properties of the targeted tissue, in addition to the materials used in the scaffold design, is essential. The development of successful 3D scaffolds requires the integration of several critical characteristics (Figure 1), including the following [95,96,97]:

(1) appropriate surface properties facilitating cell attachment, proliferation and differentiation (surface energy, chemistry, charge, surface area);
(2) a three-dimensional, highly interconnected porous network, together with an appropriate porosity, pore size and pore structure for cell growth and the transport of nutrients and metabolic waste;
(3) biocompatibility and adhesion to ensure that scaffolds integrate seamlessly with native tissues without eliciting adverse immune responses;
(4) appropriate mechanical properties that are similar to the tissue in the immediate surroundings of the defect (elastic modulus, flexural modulus, tensile strength, maximum strain);
(5) a controlled biodegradability and degradation rate that matches the rate of tissue regeneration, minimizing the risk of inflammation or structural failure;
(6) sterilization capability—compatibility with sterilization methods such as ethylene oxide, gamma radiation, UV irradiation, or ethanol, ensuring the scaffold is free from contaminants before use.

An important challenge in tissue engineering lies in designing scaffolds that mimic the architecture of native tissues. Polymeric scaffolds exhibit exceptional potential in this regard due to their tunable mechanical properties and a broad range of degradation rates, which make them suitable for diverse tissue engineering applications. By combining precise structural design with appropriate biochemical cues, scaffolds can facilitate cell attachment, proliferation, and differentiation while promoting the transport of nutrients and metabolic waste. These features position polymeric scaffolds as indispensable tools in advancing the field of regenerative medicine [96,97].

A microporous architecture of melatonin/polycaprolactone scaffolds, formed when a solution was solidified on a rolling tube (3D multilayer molding method), was characterized by a rough surface, a multilayered structure with a 0.47 μm thickness, and a mean elastic modulus of 47.06 MPa, which together could provide long-term support for peripheral nerve regrowth [81]. Additionally, after the addition of melatonin to polycaprolactone, the antioxidant and anti-inflammatory potential was greatly enhanced, along with the morphological, functional and neural recovery of the rats’ sciatic nerve.

Multilayered scaffolds composed of a polycaprolactone outer layer and an inner layer consisting of a melatonin-loaded alginate hydrogel were developed and proposed for the same application (sciatic nerve regeneration) as the scaffolds described above [92]. The materials exhibited suitable mechanical strength and excellent biocompatibility, making them promising for sciatic nerve regeneration. These scaffolds allow for controlled melatonin release, which facilitates the restoration of an ECM-like microenvironment, which is essential for effective nerve regeneration [92].

The materials based on polycaprolactone or polycaprolactone and β-Tricalcium phosphate loaded with melatonin nanoparticles showed controlled drug release for 20–22 days, with both the diffusion and dissolution mechanisms of melatonin release being revealed due to the highly interlinked porous scaffold structure (diameter 50–300 μm) [82,83]. Thanks to the controlled drug release, the in vitro evaluation of scaffolds with human chondrocytes showed increased glycosaminoglycan (GAG) deposition, which enhanced the therapeutic potential of the engineered construct in cartilage regeneration [82]. In turn, MC3T3-E1 and b.End3 cell cultures demonstrated robust growth and displayed substantial proliferation at 1, 4, and 7 days, with melatonin-loaded polycaprolactone/β-Tricalcium phosphate scaffolds for diabetic bone defect repair being tested [83]. On the other hand, melatonin-loaded magnesium–polycaprolactone scaffolds could offer therapeutics for osteosarcoma; they inhibited the proliferation, invasion and metastasis of osteosarcoma cells through the cell-in-cell pathway, which was proved in vivo and in vitro [84].

Materials based on polycaprolactone (PCL) or polylactide (PLA) enriched with natural polymers, such as sodium alginate or gelatin, have shown potential when combined with melatonin and prepared using the electrospinning technique. These composite scaffolds have demonstrated enhanced biological activity in specific applications. In the case of gelatin-enriched PCL scaffolds, an increase in PC12 cell proliferation was observed, indicating their suitability for nerve repair applications [86]. Similarly, PCL scaffolds modified with sodium alginate promoted the proliferation of tendon-derived stem cells (TDSCs), making them a viable option for tendon regeneration [20]. In both cases, porous structures were obtained, although their morphology differed significantly. This reveals that such scaffolds can provide effective nutrient delivery and drug transport to cells, which is advantageous for tissue regeneration [20,86]. Electrospun scaffolds based on PLA and gelatin, enriched with melatonin, were developed as an osteoinductive implant for bone defect repair; these exhibited an interconnected porous structure with favorable mechanical properties, which together supported cell proliferation and the osteogenic differentiation of BMMSCs (bone marrow mesenchymal stem cells) [85].

Alginate/chitosan/β-TCP composites loaded with melatonin have been obtained by the lyophilization method and used as scaffolds for bone regeneration, especially for regenerating tibia bone defects in New Zealand white rabbits (*Oryctolagus cuniculus*) [89] and for periodontal regeneration in mongrel dogs [88]. Alg/CTS/β-TCP/Mel scaffolds demonstrated a porous structure with pores that were distributed throughout the entire framework, a porosity value of about 83.64 ± 1.2%, final swelling after 2 weeks 85 ± 3.8%, an ultimate compressive strain of 26.4 ± 0.52%, an ultimate compressive strength of 92.6 ± 6.3 MPa, and a Young’s modulus of 144.3 ± 9.5 MPa. Furthermore, all the composite scaffolds retained a flat sheet shape and had not shattered randomly by the end of the compression testing [88,89]. The composite scaffolds reduced the size and depth of the defective/implanted site of the rabbit tibia bone defects across the 8-week duration [89]. In terms of the periodontal treatment of dogs, materials modified with melatonin showed accelerated bone formation and advanced maturity, with a significant twofold increase in newly formed inter-radicular bone compared with the unloaded composite. Melatonin-loaded Alg/CTS/β-TCP scaffolds allowed the complete periodontal regeneration of dogs. Furthermore, the scaffold prevented the overgrowth and entrapment of epithelial cells in furcation defects [88].

The combination of chitosan and hydroxyapatite with the addition of melatonin has been extensively studied [87,93,94]. Jarrar et al. developed and obtained chitosan/hydroxyapatite (HAp) scaffolds with two factors—melatonin and bone morphogenetic protein 2 (BMP-2)—that exert a dual function in enhancing bone formation and inhibiting osteoclast activity [87]. In the subsequent experiment, the same system was applied, but melatonin and BMP-2 were encapsulated in PLGA microparticles to enable controlled release [94]. In both cases, the materials were obtained by the lyophilization method, and they were characterized by a porous and interconnected structure with a relatively large pore size (100–200 µm), facilitating the accommodation of cells onto scaffolds by offering a high surface area and aiding in the transport of nutrients through the scaffold. Chitosan/hydroxyapatite scaffolds can be considered for bone regeneration, especially when there is a risk of osteoclast resorption, due to their osteogenic cell-differentiating and osteoclast-suppressing properties [87,94].

Melatonin-loaded gelatin porous structures have been obtained by the foaming method combined with lyophilization [91]. The sponges had good mechanical properties, good water uptake, and good water retention capacities. Improved scar formation, characterized by enhanced collagen orientation and maturity, was observed following wound treatment with gelatin-based melatonin sponges, attributed to the delivery of melatonin. Additionally, the examined materials enhanced the development of epidermal cells and sped up the repair of skin defects without adhering to the wounds [91].

As biopolymer scaffolds for wound treatment, blends of chitosan and collagen modified with melatonin have also been proposed. Scaffolds based on chitosan with marine and rat-derived collagen [23], as well as chitosan–collagen scaffolds crosslinked with glyoxal, have been studied [23]. The topical and transepidermal delivery of melatonin is a promising area for exploration in future preventive and therapeutic approaches to skin tissue engineering and wound healing. The data contributed by the experiments of Kaczmarek-Szczepańska et al. provide new insights into improvements in wound dressing where applied melatonin accelerates wound healing potential [22,23]. Thanks to the release of melatonin from chitosan/collagen scaffolds, a pronounced elevation of cell viability within human epidermal keratinocytes (NHEKs), dermal fibroblasts (NHDFs), and reference melanoma cells has been observed.

Table 2 summarizes the properties of each referenced material based on polymers enriched with melatonin. It provides a consolidated overview of the key characteristics of the polymer–melatonin-based scaffolds examined in the literature. The table lists six fundamental properties from Figure 1 and indicates whether each material composition meets these criteria. A checkmark denotes the fulfillment of a given property, allowing for a clear and efficient comparison of different scaffold formulations. The notation ‘n/a’ signifies that no information on a particular property is available in the referenced study or that the property is not applicable to the material in question.

**Table 2:** A summary of the key characteristics of the polymer–melatonin-based scaffolds reported in the literature.

Matrix	(1) Appropriate Surface Properties	(2) Porous Structure	(3) Biocompatibility	(4) Appropriate Mechanical Properties	(5) Controlled Biodegradability and Degradation Rate with Controlled Drug Release	(6) Sterilization Capability	References
Polycaprolactone	n/a	✓	✓	✓	✓	n/a	[81,82]
Gelatine	n/a	✓	✓	✓	✓	✓	[91]
Magnesium/ polycaprolactone	✓	✓	✓	✓	✓	n/a	[84]
Polycaprolactone/ β-TCP/PLGA NPs	n/a	✓	✓	✓	✓	n/a	[83]
polycaprolactone/sodium alginate	✓	✓	✓	✓	✓	✓	[20,92]
Polycaprolactone/gelatin	✓	✓	✓	✓	✓	n/a	[86]
Gelatin/ polylactide	n/a	✓	✓	✓	✓	n/a	[85]
Alginate/ chitosan/β-TCP	n/a	✓	✓	✓	n/a	✓	[88,89]
Chitosan/ hydroxyapatite	n/a	✓	✓	✓	✓	n/a	[87,93]
Chitosan/ hydroxyapatite/ PLGA NPs	n/a	✓	✓	✓	✓	✓	[94]
Chitosan/ collagen	n/a	✓	✓	✓	✓	n/a	[22,23]

✓—the material meets the criteria; n/a—no information on a particular property is available in the referenced study or the property is not applicable to the material.

## 6. Conclusions

Integrating melatonin into biopolymeric scaffolds presents exciting opportunities for advancements in tissue engineering. Despite its therapeutic promise, challenges such as maintaining melatonin’s stability under exposure to light, heat, and oxygen persist. Strategies such as encapsulation in nanoparticles, protective coatings, or embedding within hydrogel matrices are being explored to shield melatonin and preserve its bioactivity during storage and application. These techniques aim to ensure the sustained release of melatonin, aligning with the specific requirements of tissue regeneration.

Future advancements in biopolymeric scaffolds with melatonin should focus on enhancing their adaptability, efficiency, and therapeutic potential. Smart, stimuli-responsive materials could enable precise melatonin release based on biological signals such as the pH, enzymatic activity, or oxidative stress, ensuring optimal delivery at the required site and time. The incorporation of nanomaterials like graphene oxide, bioactive glass, and carbon nanotubes may further improve the mechanical strength, electrical conductivity, and bioactivity, making scaffolds more effective for nerve and musculoskeletal tissue regeneration.

The integration of bioprinting technology and AI-driven computational modeling could lead to patient-specific scaffolds with an optimized architecture and porosity, enhancing cell adhesion, proliferation, and differentiation while ensuring controlled melatonin release. Scaffold fabrication may also evolve to support dual or multi-phase drug delivery, where melatonin is combined with growth factors such as platelet-derived growth factor (PDGF) or vascular endothelial growth factor (VEGF), allowing for a time-controlled therapeutic response tailored to different stages of tissue healing.

Electrostimulation therapy could be enhanced through the development of bioelectronic scaffolds incorporating electrically conductive biopolymers, improving cellular communication and differentiation, particularly in nerve regeneration. Additionally, injectable melatonin-loaded hydrogel scaffolds could enable minimally invasive tissue engineering approaches, forming gel-like structures in situ to conform to tissue defects while maintaining sustained melatonin release.

Future scaffold designs are evolving toward “smart scaffolds”, which can dynamically respond to environmental cues such as pH, temperature, or mechanical stress. This adaptability could significantly enhance the therapeutic application of melatonin by providing context-specific release profiles. Furthermore, combining melatonin with complementary bioactive molecules such as growth factors, cytokines, or antimicrobial agents may lead to multifunctional scaffolds that are capable of addressing complex regeneration scenarios. These combinations could synergistically enhance key processes, including angiogenesis, inflammation modulation, and microbial resistance, further optimizing the healing environment.

Innovative scaffold fabrication techniques such as 3D printing, electrospinning, and lyophilization provide tailored solutions for incorporating melatonin. These methods allow precise control over scaffold architecture, porosity, and drug delivery profiles, ensuring effective interaction with target tissues. For instance, melatonin-loaded polycaprolactone scaffolds have demonstrated promising results in both bone and nerve regeneration, showing controlled release and enhanced cell proliferation.

## References

[1] Ebhodaghe S.O. (2021). Natural Polymeric Scaffolds for Tissue Engineering Applications. J. Biomater. Sci. Polym. Ed..

[2] Zarrintaj P., Seidi F., Azarfam M.Y., Yazdi M.K., Erfani A., Barani M., Chauhan N.P.S., Rabiee N., Kuang T., Kucinska-Lipka J. (2023). Biopolymer-based composites for tissue engineering applications: A basis for future opportunities. Compos. B Eng..

[3] Behrens M.R., Ruder W.C. (2021). Biopolymers in Regenerative Medicine: Overview, Current Advances, and Future Trends. Biopolymers for Biomedical and Biotechnological Applications.

[4] Veeman D., Sai M.S., Sureshkumar P., Jagadeesha T., Natrayan L., Ravichandran M., Mammo W.D. (2021). Additive Manufacturing of Biopolymers for Tissue Engineering and Regenerative Medicine: An Overview, Potential Applications, Advancements, and Trends. Int. J. Polym. Sci..

[5] Danglad-Flores J., Sletten E.T., Reuber E.E., Bienert K., Riegler H., Seeberger P.H. (2024). Optimized platform for automated glycan assembly. Device.

[6] Guo B., Lei B., Li P., Ma P.X. (2015). Functionalized scaffolds to enhance tissue regeneration. Regen. Biomater..

[7] John J.V., McCarthy A., Karan A., Xie J. (2022). Electrospun Nanofibers for Wound Management. ChemNanoMat.

[8] Grayson W.L., Martens T.P., Eng G.M., Radisic M., Vunjak-Novakovic G. (2009). Biomimetic approach to tissue engineering. Semin. Cell Dev. Biol..

[9] Xia P., Luo Y. (2022). Vascularization in tissue engineering: The architecture cues of pores in scaffolds. J. Biomed. Mater. Res. B Appl. Biomater..

[10] Lekhavadhani S., Shanmugavadivu A., Selvamurugan N. (2023). Role and architectural significance of porous chitosan-based scaffolds in bone tissue engineering. Int. J. Biol. Macromol..

[11] Farazin A., Zhang C., Gheisizadeh A., Shahbazi A. (2023). 3D bio-printing for use as bone replacement tissues: A review of biomedical application. Biomed. Eng. Adv..

[12] Mukasheva F., Adilova L., Dyussenbinov A., Yernaimanova B., Abilev M., Akilbekova D. (2024). Optimizing scaffold pore size for tissue engineering: Insights across various tissue types. Front. Bioeng. Biotechnol..

[13] Camacho P., Busari H., Seims K.B., Schwarzenberg P., Dailey H.L., Chow L.W. (2019). 3D printing with peptide–polymer conjugates for single-step fabrication of spatially functionalized scaffolds. Biomater. Sci..

[14] Rahmany M.B., Van Dyke M. (2013). Biomimetic approaches to modulate cellular adhesion in biomaterials: A review. Acta Biomater..

[15] Tai Y., Banerjee A., Goodrich R., Jin L., Nam J. (2021). Development and Utilization of Multifunctional Polymeric Scaffolds for the Regulation of Physical Cellular Microenvironments. Polymers.

[16] Wang X., He M., Wang X., Liu S., Luo L., Zeng Q., Wu Y., Zeng Y., Yang Z., Sheng G. (2024). Emerging Nanochitosan for Sustainable Agriculture. Int. J. Mol. Sci..

[17] Sohn E.-H., Kim S.-N., Lee S.-R. (2024). Melatonin’s Impact on Wound Healing. Antioxidants.

[18] Murali R., Thanikaivelan P., Cheirmadurai K. (2016). Melatonin in functionalized biomimetic constructs promotes rapid tissue regeneration in Wistar albino rats. J. Mater. Chem. B.

[19] Xu Y., Chen X., Qian Y., Tang H., Song J., Qu X., Yue B., Yuan W. (2020). Melatonin-Based and Biomimetic Scaffold as Muscle–ECM Implant for Guiding Myogenic Differentiation of Volumetric Muscle Loss. Adv. Funct. Mater..

[20] Yao Z., Qian Y., Jin Y., Wang S., Li J., Yuan W.-E., Fan C. (2022). Biomimetic multilayer polycaprolactone/sodium alginate hydrogel scaffolds loaded with melatonin facilitate tendon regeneration. Carbohydr. Polym..

[21] Aykora D., Oral A., Aydeğer C., Uzun M. (2025). 3D Bioprinting Strategies for Melatonin-Loaded Polymers in Bone Tissue Engineering. Macromol. Mater. Eng..

[22] Kaczmarek-Szczepańska B., Pin J.M., Zasada L., Sonne M.M., Reiter R.J., Slominski A.T., Steinbrink K., Kleszczyński K. (2022). Assessment of Melatonin-Cultured Collagen/Chitosan Scaffolds Cross-Linked by a Glyoxal Solution as Biomaterials for Wound Healing. Antioxidants.

[23] Kaczmarek-Szczepańska B., Ostrowska J., Kozłowska J., Szota Z., Brożyna A.A., Dreier R., Reiter R.J., Slominski A.T., Steinbrink K., Kleszczyński K. (2021). Evaluation of Polymeric Matrix Loaded with Melatonin for Wound Dressing. Int. J. Mol. Sci..

[24] Franco P.I.R., do Carmo Neto J.R., Rocha V.L., Machado J.R., Amaral A.C., Miguel M.P. (2023). A Revision of Polymeric Nanoparticles as a Strategy to Improve the Biological Activity of Melatonin. Curr. Med. Chem..

[25] Chuffa L.G.d.A., Seiva F.R.F., Novais A.A., Simão V.A., Giménez V.M.M., Manucha W., Zuccari D.A.P.d.C., Reiter R.J. (2021). Melatonin-Loaded Nanocarriers: New Horizons for Therapeutic Applications. Molecules.

[26] Adamiak K., Gaida V.A., Schäfer J., Bosse L., Diemer C., Reiter R.J., Slominski A.T., Steinbrink K., Sionkowska A., Kleszczyński K. (2024). Melatonin/Sericin Wound Healing Patches: Implications for Melanoma Therapy. Int. J. Mol. Sci..

[27] Biswal T. (2021). Biopolymers for tissue engineering applications: A review. Mater. Today Proc..

[28] Ullah S., Chen X. (2020). Fabrication, applications and challenges of natural biomaterials in tissue engineering. Appl. Mater. Today.

[29] Jabbari E. (2019). Challenges for Natural Hydrogels in Tissue Engineering. Gels.

[30] Asghar M.S., Li J., Ahmed I., Ghazanfar U., Irshad M.S., Idrees M., Haq Z., Rizwan M., Sheikh F., Yasmeen F. (2021). Antioxidant, and enhanced flexible nano porous scaffolds for bone tissue engineering applications. Nano Sel..

[31] Selvakumar G., Lonchin S. (2023). A bio-polymeric scaffold incorporated with p-Coumaric acid enhances diabetic wound healing by modulating MMP-9 and TGF-β3 expression. Colloids Surf. B Biointerfaces.

[32] Karan A., Sharma N.S., Darder M., Su Y., Andrabi S.M., Shahriar S.M.S., John J.V., Luo Z., DeCoster M.A., Zhang Y.S. (2024). Copper–Cystine Biohybrid-Embedded Nanofiber Aerogels Show Antibacterial and Angiogenic Properties. ACS Omega.

[33] John J.V., Sharma N.S., Tang G., Luo Z., Su Y., Weihs S., Shahriar S.M.S., Wang G., McCarthy A., Dyke J. (2023). Nanofiber Aerogels with Precision Macrochannels and LL-37-Mimic Peptides Synergistically Promote Diabetic Wound Healing. Adv. Funct. Mater..

[34] Rice J.J., Martino M.M., De Laporte L., Tortelli F., Briquez P.S., Hubbell J.A. (2013). Engineering the Regenerative Microenvironment with Biomaterials. Adv. Healthc. Mater..

[35] López-Gutierrez J., Ramos-Payán R., Ayala-Ham A., Romero-Quintana J.G., Castillo-Ureta H., Villegas-Mercado C., Bermúdez M., Sanchez-Schmitz G., Aguilar-Medina M. (2023). Biofunctionalization of hydrogel-based scaffolds for vascular tissue regeneration. Front. Mater..

[36] Casanova M.R., Reis R.L., Martins A., Neves N.M. (2020). Surface biofunctionalization to improve the efficacy of biomaterial substrates to be used in regenerative medicine. Mater. Horiz..

[37] Boddepalli Y., Chava N., Gadiraju S., Sri Pachchava K., Kotikalapudi K., Siddiqui N. (2023). Review on Growth Factor Loaded Scaffolds for Rapid Healing of Bone Tissue. Int. J. Life Sci. Pharma Res..

[38] Safari B., Davaran S., Aghanejad A. (2021). Osteogenic potential of the growth factors and bioactive molecules in bone regeneration. Int. J. Biol. Macromol..

[39] De Witte T.-M., Fratila-Apachitei L.E., Zadpoor A.A., Peppas N.A. (2018). Bone tissue engineering via growth factor delivery: From scaffolds to complex matrices. Regen. Biomater..

[40] Barabaschi G.D.G., Manoharan V., Li Q., Bertassoni L.E. (2015). Engineering Pre-Vascularized Scaffolds for Bone Regeneration.

[41] Davies N.H., Schmidt C., Bezuidenhout D., Zilla P. (2012). Sustaining Neovascularization of a Scaffold Through Staged Release of Vascular Endothelial Growth Factor-A and Platelet-Derived Growth Factor-BB. Tissue Eng. Part A.

[42] Karpov T.E., Peltek O.O., Muslimov A.R., Tarakanchikova Y.V., Grunina T.M., Poponova M.S., Karyagina A.S., Chernozem R.V., Pariy I.O., Mukhortova Y.R. (2020). Development of Optimized Strategies for Growth Factor Incorporation onto Electrospun Fibrous Scaffolds to Promote Prolonged Release. ACS Appl. Mater. Interfaces.

[43] Seims K.B., Hunt N.K., Chow L.W. (2021). Strategies to Control or Mimic Growth Factor Activity for Bone, Cartilage, and Osteochondral Tissue Engineering. Bioconjug. Chem..

[44] Bayer E.A., Gottardi R., Fedorchak M.V., Little S.R. (2015). The scope and sequence of growth factor delivery for vascularized bone tissue regeneration. J. Control. Release.

[45] Kass L.E., Nguyen J. (2021). Nanocarrier-hydrogel composite delivery systems for precision drug release. WIREs Nanomed. Nanobiotechnol..

[46] Rahman S.U., Nagrath M., Ponnusamy S., Arany P.R. (2018). Nanoscale and Macroscale Scaffolds with Controlled-Release Polymeric Systems for Dental Craniomaxillofacial Tissue Engineering. Materials.

[47] Ali F., Khan I., Chen J., Akhtar K., Bakhsh E.M., Khan S.B. (2022). Emerging Fabrication Strategies of Hydrogels and Its Applications. Gels.

[48] Yanez M., Blanchette J., Jabbarzadeh E. (2018). Modulation of Inflammatory Response to Implanted Biomaterials Using Natural Compounds. Curr. Pharm. Des..

[49] Julier Z., Park A.J., Briquez P.S., Martino M.M. (2017). Promoting tissue regeneration by modulating the immune system. Acta Biomater..

[50] Batool F., Özçelik H., Stutz C., Gegout P.-Y., Benkirane-Jessel N., Petit C., Huck O. (2021). Modulation of immune-inflammatory responses through surface modifications of biomaterials to promote bone healing and regeneration. J. Tissue Eng..

[51] Muzzio N., Moya S., Romero G. (2021). Multifunctional Scaffolds and Synergistic Strategies in Tissue Engineering and Regenerative Medicine. Pharmaceutics.

[52] Todd E.A., Mirsky N.A., Silva B.L.G., Shinde A.R., Arakelians A.R.L., Nayak V.V., Marcantonio R.A.C., Gupta N., Witek L., Coelho P.G. (2024). Functional Scaffolds for Bone Tissue Regeneration: A Comprehensive Review of Materials, Methods, and Future Directions. J. Funct. Biomater..

[53] Hunziker R., Lumelsky N., Wang F. (2015). Editorial: Scaffolds for Regenerative Medicine: A Special Issue of the Annals of Biomedical Engineering. Ann. Biomed. Eng..

[54] Ali Zahid A., Chakraborty A., Shamiya Y., Ravi S.P., Paul A. (2022). Leveraging the advancements in functional biomaterials and scaffold fabrication technologies for chronic wound healing applications. Mater. Horiz..

[55] Oliveira R.L.M.S., Barbosa L., Hurtado C.R., Ramos L.d.P., Montanheiro T.L.A., Oliveira L.D., Tada D.B., Trichês E.d.S. (2020). Bioglass-based scaffolds coated with silver nanoparticles: Synthesis, processing and antimicrobial activity. J. Biomed. Mater. Res. A.

[56] Kalyan K.S.D.R., Vinay C., Arunbhupathi, Uloopi K.S., Chandrasekhar R., RojaRamya K.S. (2019). Preclinical Evaluation and Clinical Trial of Chlorhexidine Polymer Scaffold for Vital Pulp Therapy. J. Clin. Pediatr. Dent..

[57] Felice B., Sánchez M.A., Socci M.C., Sappia L.D., Gómez M.I., Cruz M.K., Felice C.J., Martí M., Pividori M.I., Simonelli G. (2018). Controlled degradability of PCL-ZnO nanofibrous scaffolds for bone tissue engineering and their antibacterial activity. Mater. Sci. Eng. C.

[58] Chen X., Zhang Q., Wang Y., Meng J., Wu M., Xu H., Du L., Yang X. (2023). Fabrication and Characterization of Electrospun Poly(Caprolactone)/Tannic Acid Scaffold as an Antibacterial Wound Dressing. Polymers.

[59] Jiménez-Gastélum G.R., Aguilar-Medina E.M., Soto-Sainz E., Ramos-Payán R., Silva-Benítez E.L. (2019). Antimicrobial Properties of Extracellular Matrix Scaffolds for Tissue Engineering. Biomed. Res. Int..

[60] Sarkar N., Bose S. (2019). Liposome-Encapsulated Curcumin-Loaded 3D Printed Scaffold for Bone Tissue Engineering. ACS Appl. Mater. Interfaces.

[61] Murphy K.P., Hendley M.A., Isely C., Annamalai P., Peña E., Gower R.M. (2018). Resveratrol Delivery from Porous Poly(lactide- co -glycolide) Scaffolds Promotes an Anti-Inflammatory Environment within Visceral Adipose Tissue. ACS Appl. Mater. Interfaces.

[62] Li X., Wang Y., Wang Z., Qi Y., Li L., Zhang P., Chen X., Huang Y. (2018). Composite PLA/PEG/nHA/Dexamethasone Scaffold Prepared by 3D Printing for Bone Regeneration. Macromol. Biosci..

[63] Marrazzo P., O’Leary C. (2020). Repositioning Natural Antioxidants for Therapeutic Applications in Tissue Engineering. Bioengineering.

[64] Jayasinghe S.N. (2017). Thoughts on Scaffolds. Adv. Biosyst..

[65] Luchetti F., Canonico B., Bartolini D., Arcangeletti M., Ciffolilli S., Murdolo G., Piroddi M., Papa S., Reiter R.J., Galli F. (2014). Melatonin regulates mesenchymal stem cell differentiation: A review. J. Pineal Res..

[66] Dalgic A.D., Atila D., Tezcaner A., Gürses S., Keskin D. (2023). Diatom silica frustules-doped fibers for controlled release of melatonin for bone regeneration. Eur. Polym. J..

[67] Reiter R.J., Mayo J.C., Tan D., Sainz R.M., Alatorre-Jimenez M., Qin L. (2016). Melatonin as an antioxidant: Under promises but over delivers. J. Pineal Res..

[68] Hardeland R. (2018). Melatonin and inflammation—Story of a double-edged blade. J. Pineal Res..

[69] Favero G., Franceschetti L., Bonomini F., Rodella L.F., Rezzani R. (2017). Melatonin as an Anti-Inflammatory Agent Modulating Inflammasome Activation. Int. J. Endocrinol..

[70] Radogna F., Diederich M., Ghibelli L. (2010). Melatonin: A pleiotropic molecule regulating inflammation. Biochem. Pharmacol..

[71] Sánchez-Barceló E.J., Mediavilla M.D., Tan D.X., Reiter R.J. (2010). Scientific Basis for the Potential Use of Melatonin in Bone Diseases: Osteoporosis and Adolescent Idiopathic Scoliosis. J. Osteoporos..

[72] Maria S., Samsonraj R.M., Munmun F., Glas J., Silvestros M., Kotlarczyk M.P., Rylands R., Dudakovic A., van Wijnen A.J., Enderby L.T. (2018). Biological effects of melatonin on osteoblast/osteoclast cocultures, bone, and quality of life: Implications of a role for MT2 melatonin receptors, MEK1/2, and MEK5 in melatonin-mediated osteoblastogenesis. J. Pineal Res..

[73] Narayan S., Malaiappan S. (2023). Additive Benefits of Melatonin on Osteogenic Differentiation Rate and Osteogenic Potential Quantified by Alkaline Phosphatase—A Systematic Review. Int. J. Biomed. Investig..

[74] Miao Y., Chen Y., Liu X., Diao J., Zhao N., Shi X., Wang Y. (2019). Melatonin decorated 3D-printed beta-tricalcium phosphate scaffolds promoting bone regeneration in a rat calvarial defect model. J. Mater. Chem. B.

[75] Majidinia M., Reiter R.J., Shakouri S.K., Mohebbi I., Rastegar M., Kaviani M., Darband S.G., Jahanban-Esfahlan R., Nabavi S.M., Yousefi B. (2018). The multiple functions of melatonin in regenerative medicine. Ageing Res. Rev..

[76] Mousavi S.M., Etemad L., Yari D., Hashemi M., Salmasi Z. (2024). Evaluation of Melatonin and its Nanostructures Effects on Skin Disorders Focused on Wound Healing. Mini-Rev. Med. Chem..

[77] Zhang H., Zhang Y. (2014). Melatonin: A well-documented antioxidant with conditional pro-oxidant actions. J. Pineal Res..

[78] Bonnefont-Rousselot D., Collin F. (2010). Melatonin: Action as antioxidant and potential applications in human disease and aging. Toxicology.

[79] Mauriz J.L., Collado P.S., Veneroso C., Reiter R.J., González-Gallego J. (2013). A review of the molecular aspects of melatonin’s anti-inflammatory actions: Recent insights and new perspectives. J. Pineal Res..

[80] Goradel N.H., Asghari M.H., Moloudizargari M., Negahdari B., Haghi-Aminjan H., Abdollahi M. (2017). Melatonin as an angiogenesis inhibitor to combat cancer: Mechanistic evidence. Toxicol. Appl. Pharmacol..

[81] Qian Y., Han Q., Zhao X., Song J., Cheng Y., Fang Z., Ouyang Y., Yuan W., Fan C. (2018). 3D melatonin nerve scaffold reduces oxidative stress and inflammation and increases autophagy in peripheral nerve regeneration. J. Pineal Res..

[82] Rao S.K., Jaison D., Sridhar K., Kasthuri N., Gopinath V., Sivaperumal P., Patil S. (2020). Melatonin delivery from PCL scaffold enhances glycosaminoglycans deposition in human chondrocytes—Bioactive scaffold model for cartilage regeneration. Process Biochem..

[83] Chen T., Wu Z., Hou Q., Mei Y., Yang K., Xu J., Wang L. (2024). The Dual Angiogenesis Effects via Nrf2/HO-1 Signaling Pathway of Melatonin Nanocomposite Scaffold on Promoting Diabetic Bone Defect Repair. Int. J. Nanomed..

[84] Zhang W., Zhao W., Li Q., Zhao D., Qu J., Yuan Z., Cheng Z., Zhu X., Zhuang X., Zhang Z. (2021). 3D-printing magnesium–polycaprolactone loaded with melatonin inhibits the development of osteosarcoma by regulating cell-in-cell structures. J. Nanobiotechnol..

[85] Lv N., Hou M., Deng L., Hua X., Zhou X., Liu H., Zhu X., Xu Y., Qian Z., Li Q. (2024). A sponge-like nanofiber melatonin-loaded scaffold accelerates vascularized bone regeneration via improving mitochondrial energy metabolism. Mater. Today Bio.

[86] Chen T., Jiang H., Li X., Zhang D., Zhu Y., Chen X., Yang H., Shen F., Xia H., Zheng J. (2022). Proliferation and differentiation study of melatonin functionalized polycaprolactone/gelatin electrospun fibrous scaffolds for nerve tissue engineering. Int. J. Biol. Macromol..

[87] Jarrar H., Çetin Altındal D., Gümüşderelioğlu M. (2021). Effect of melatonin/BMP-2 co-delivery scaffolds on the osteoclast activity. J. Mater. Sci. Mater. Med..

[88] Abdelrasoul M., El-Fattah A.A., Kotry G., Ramadan O., Essawy M., Kamaldin J., Kandil S. (2023). Regeneration of critical-sized grade II furcation using a novel injectable melatonin-loaded scaffold. Oral. Dis..

[89] Abdelrasoul M.R., Kamaldin J., Ooi J.P., El-Fattah A.A., Kotry G.S., Ramadan O., Kandil S. (2020). An Eight-Week In Vivo Study on the Clinical Signs of Systemic Toxicity and Bone Regenerative Performance of Composites Containing Beta Tricalcium Phosphate, Hydrogel and Melatonin in Adult New Zealand Rabbit (*Oryctolagus cuniculus*). Malays. J. Med. Health Sci..

[90] Perić Kačarević Ž., Rider P., Alkildani S., Retnasingh S., Pejakić M., Schnettler R., Gosau M., Smeets R., Jung O., Barbeck M. (2020). An introduction to bone tissue engineering. Int. J. Artif. Organs.

[91] Chanu N.R., Bhattacharya K., Marbaniang D., Pal P., Ray S., Mazumder B. (2022). Evaluation of a novel melatonin-loaded gelatin sponge as a wound dressing. J. Vasc. Nurs..

[92] Wang X., Yao X., Sun Z., Jin Y., Yan Z., Jiang H., Ouyang Y., Yuan W.-E., Wang C., Fan C. (2023). An extracellular matrix mimicking alginate hydrogel scaffold manipulates an inflammatory microenvironment and improves peripheral nerve regeneration by controlled melatonin release. J. Mater. Chem. B.

[93] Çetin Altındal D., Gümüşderelioğlu M. (2019). Dual-functional melatonin releasing device loaded with PLGA microparticles and cyclodextrin inclusion complex for osteosarcoma therapy. J. Drug Deliv. Sci. Technol..

[94] Jarrar H., Çetin Altındal D., Gümüşderelioğlu M. (2021). Scaffold-based osteogenic dual delivery system with melatonin and BMP-2 releasing PLGA microparticles. Int. J. Pharm..

[95] Suamte L., Tirkey A., Barman J., Jayasekhar Babu P. (2023). Various manufacturing methods and ideal properties of scaffolds for tissue engineering applications. Smart Mater. Manuf..

[96] Dhandayuthapani B., Yoshida Y., Maekawa T., Kumar D.S. (2011). Polymeric Scaffolds in Tissue Engineering Application: A Review. Int. J. Polym. Sci..

[97] Sultana N. (2013). Scaffolds for Tissue Engineering.

